# Malaria in Mazandaran, Northern Iran: Passive Case Finding During 1997-2012

**Published:** 2012

**Authors:** S Ghaffari, SA Mahdavi, Z Moulana, S Mouodi, H Karimi-Nia, M Bayani, N Kalantari

**Affiliations:** 1 Department of Parasitology and Mycology, Faculty of Medicine, Babol University of Medical Sciences, Babol, Iran; 2 Health Center, Mazandaran University of Medical Sciences, Sari, Iran; 3 Department of Laboratory Sciences, Faculty of Para-Medicine, Babol University of Medical Sciences, Babol, Iran; 4 Health Center, Babol University of Medical Sciences, Babol, Iran; 5 Health Center, Babolsar, Iran; 6 Infection and Tropical Disease Research Center; Infectious Diseases Department, Faculty of Medicine; Babol University of Medical Sciences, Babol, Iran; 7 Cellular and Molecular Research Center, Babol University of Medical Sciences; Department of Laboratory Sciences, Faculty of Para-Medicine; Babol University of Medical Sciences, Babol, Iran

**Keywords:** Malaria, *P. falciparum*, *P. vivax*, Iran

## Abstract

**Background:**

Malaria is one of the most important parasitic diseases in tropical and temperate regions. The aim of this study was to determine the trend of malaria in Mazandaran Province, northern Iran during 1997-2012.

**Methods:**

This retrospective study was conducted from 1997 to 2012. The population's study was individuals who registered at health centers of Mazandaran Province. Peripheral blood smear were prepared for each case, stained with Giemsa and examined by light microscope. In addition to demographic data, other parameters including Slide Positive Rate (SPR), Annual Parasite Incidence (API) and Annual Blood Examination Rate (ABER) were analyzed.

**Results:**

In total, 844 cases of malaria were reported. *Plasmodium vivax* was predominant species with 821 cases (97.4%). The number of malaria cases increased from 1997 to 2005 and then decreased to 3 cases in 2011. Some cities had not reported any cases during last three years. The highest infection rate, 163(20.07%), was seen in 2001-02. The SPR had the highest value (0.54%) in 2004-05. The maximum API and ABER were observed in 2001-02 and 1997-98. 641(75.9%) of cases were imported from hyperendemic areas such as Afghanistan and South-eastern Iran and 94 (11.1%) malaria patients were recorded as introduced cases. The highest infection rate of malaria (21.3%) was seen in Babolsar.

**Conclusion:**

Extensive malaria control should be continued to Mazandaran to become malaria-free region and in prevention of re-introduction stage.

## Introduction

Malaria is still one of the most important infectious diseases in the world, mainly in the developing countries. Approximately half of the world's populations are currently in potential risk of contracting malaria. About 250 million new cases of the disease diagnosed and one million people die from malaria annually in the world (1). High morbidity and mortality rate of malaria are seen in Sub-Saharan Africa constitutes (2). However, approximately 60% of the Eastern Mediterranean Region's populations are also at risk of malaria. Iran is one of the countries placed in this area which malaria endemicity is low in some of its regions (2–4).

Based on epidemiological conditions, Iran had been divided to three different regions with various endemicity of malaria in the past and nowadays, according to the National Strategy Plan for Malaria Control, the entire country has been divided into four sections (3). Northern Iran, the littoral plain of Caspian Sea and forest areas of north slops of Alborz Mountains, which has Mediterranean weather with 800-1200 mm annual rainfall, 70%-100% relative humidity, and 10-35 °C average temperature, had been considered as a malaria hyper endemic region before 1950 (3). *Plasmodium vivax* was the prevalent species and *Anopheles maculipennis* was malaria vector. National malaria control program's was carried out during 1950-79s lead to nearly interrupted disease transmission and almost eliminated malaria in the north parts of the country (2, 3). Unfortunately, malaria re-emerges in northern Iran in 1994 after a large displacement of people from the Republic of Azerbaijan and to some extent from Armenia (5). Now, it is considered as an area where the imported cases are found and the potential risk of malaria transmission exists (3).

Malaria was problematic in Mazandaran Province, like other parts of northern Iran, in the past and at the moment it is under some potential risk of transmission. In Mazandaran the prevalence and incidence rates of malaria are vary during different years. Researchers reported 3291 cases from there and the number of positive cases was decreased gradually to 66 in 1993 and then increased (6). The number of malaria cases and annual parasite incidence (API) rate increased from 66 and 0.02/1000 in 1999 to 129 and 0.05/1000 in 2003, respectively (7).

However, Mazandaran has particular geographical condition and has tourist's attractions and hospitality for refugees. Furthermore, movement of people from different parts of Iran and other countries to this province for holidays, job-seeking and financial activities may affect parasite populations and cause the re-introduction of malaria into this region (3, 5). Hence, regularly monitoring should be performed in there. For that reason, according with nearly all malaria control programs in the world and near all studies performed on malaria based on passive case detection (PCD), the present study was carried out to find the current malaria situation in Mazandaran Province during 1997-2012.

## Materials and Methods

### Study area

**Fig. 1:**
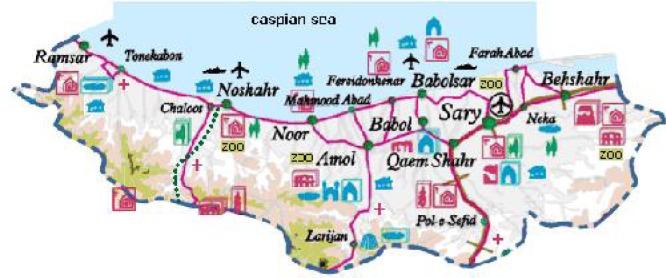
Map of Mazandaran Province (http://tabarestan.4t.com/english-map.htm)

This retrospective study was conducted in the Mazandaran Province, northern Iran from 1997 to 2012. The area has 23,831 km^2^, closed to Caspian Sea and at the interface of the Golestan, Gilan and Tehran provinces from northeast, northwest and south, respectively. Therefore, it has important role in replacement of populations and travelers. This province has diverse climate conditions and region situations (hilly, valley and plain regions) and consists of 16 cities and 102 rural districts (Fig. 1). Mean annual temperature is varying between 12.5 to 20°C, while annual rainfall ranges are from 718 to 1274 mm. The rainy season peaks between September and January, while the driest months are between June and August (8).

### Study population

The population's study was all suspected cases attended to Health centers of the province. Peripheral blood smear were prepared for each case, stained with Giemsa and examined by light microscope. The results obtained by microscopy and demographic information were accurately recorded. In addition to demographic data, other parameters including the slide positive rate (SPR), the annual parasite incidence (API) and the annual blood examination rate (ABER) were analyzed (9).

The API was calculated as the incidence of malaria per 1,000 inhabitants (9–10) and the SPR was expressed as the number of positive slides/total number of slides examined (11).

The study was approved by the regional Ethics Committee of the Research Department of Babol University of Medical Sciences, Babol, Iran. Data were analyzed by SPSS V.18.

## Results

Of the 470,424 peripheral blood smear examined microscopically during 1997-2012, 844 (0.18%) were positive for malaria parasites. Of these, 822 (97.4%) were due to *Plasmodium vivax*, 16(1.9%) were due to *P. falciparum* and 1(0.1%) was due to *P. malariae*. In addition, 3 (0.4%) cases had mixed infection and species was not identified in 2 (0.2%) patients (Table 1).

**Table 1:** Prevalence of *Plasmodium* species, slide positive rate (SPR), annual parasite incidence (API) and annual blood examination rate (ABER) in Mazandaran Province, Iran, 1997-2012

Year	Number of			*Plasmodium*					SPR	API	ABER
	Population	Slide	Positive	*vivax*	*falciparum*	*malariae*	Mixed	Unknown			
1997-8	2,632,191	64,420	58	54	-	1	1	2	0.09	0.022	24.47
1998-9	2,662,724	45,676	54	54	-	-	-	-	0.12	0.020	17.15
1999-2000	2,693,612	41,739	59	55	4	-	-	-	0.14	0.021	15. 5
2000-01	2,724,858	38,407	60	58	2	-	-	-	0.16	0.022	14.1
2001-02	2,756,466	42,386	163	161	2	-	-	-	0.38	0.059	15.39
2002-03	2,788,441	41,214	131	127	4	-	-	-	0.32	0.046	14.78
2003-04	2,820,787	22,603	83	82	1	-	-	-	0.37	0.029	8.013
2004-05	2,853,508	28,199	153	150	1	-	2	-	0.54	0.053	9.882
2005-06	2,886,609	23,381	40	40	-	-	-	-	0.17	0.013	8.099
2006-07	2,922,432	21,232	12	10	2	-	-	-	0.05	0.004	7.265
2007-08	2,951,687	22425	3	3	-	-	-	-	0.01	0.001	7.597
2008-09	2,985,927	21364	11	11	-	-	-	-	0.05	0.004	7.154
2009-2010	3,020,563	21554	10	10	-	-	-	-	0.05	0.003	7.135
2010-11	3,055,602	20461	4	4	-	-	-	-	0.02	0.001	6.696
2011-12	3,091,047	15363	3	3	-	-	-	-	0.02	0.001	4.9701
**Total**	**Number** 470424		844	821	16	1	3	2			
	**Percentage**		100	97.4	1.9	0.1	0.4	0.2			

The number of malaria cases and the SPR varied in early years but it was almost constant during 2007-2012. The highest peak of malaria cases occurred in 2001-2002 with 163 cases and slightly decreased in the two next years and then increased to 153 cases in 2004-2005. The highest amount of SPR (0.54%) was seen in 2004-2005. Other information's such as SPR, API and ABER were also shown in Table 1. Moreover, data about the number of cases imported from other countries or other provinces, and introduced cases are shown in Table 2. The proportion cases considered imported from other provinces (B), or those found in foreign nationals (A), varied greatly over the years.

**Table 2:** Frequency of malaria cases in associated with some epidemiological data, Mazandaran province, Iran, 1997-2012. (A is imported cases from other countries and B is imported cases from other provinces)

year	A		B		Introduced		Local		Unknown		Relapse		Total	
	N	%	N	%	N	%	N	%	N	%	N	%	N	%
**1997-1998**	24	41.4	4	6.9	0	0	13	22.4	7	12.1	10	17.2	58	100
**1998-1999**	24	44.4	2	3.7	0	0	0	0	14	25.9	14	25.9	54	100
**1999-2000**	48	81.4	5	8.5	4	6.7	0	0	0	0	2	3.4	59	100
**2000-2001**	49	81.7	1	1.7	7	11.6	0	0	2	3.3	1	1.7	60	100
**2001-2002**	130	79.8	4	2.5	28	17.2	0	0	1	0.6		0	163	100
**2002-2003**	90	68.7	3	2.3	32	24.4	0	0	4	3.1	2	1.5	131	100
**2003-2004**	78	94	2	2.4	2	2.4	0	0	1	1.2	0	0	83	100
**2004-2005**	138	90.2	6	3.9	6	3.9	0	0	3	2	0	0	153	100
**2005-2006**	26	65	0	0	13	32.5	0	0	0	0	1	2.5	40	100
**2006-2007**	10	83.3	0	0	0	0	0	0	2	16.7	0	0	12	100
**2007-2008**	1	33.3	0	0	2	66.7	0	0	0	0	0	0	3	100
**2008-2009**	10	90.9	1	9.1	0	0	0	0	0	0	0	0	11	100
**2009-2010**	8	80	2	20	0	0	0	0	0	0	0	0	10	100
**2010-2011**	3	75	1	25	0	0	0	0	0	0	0	0	4	100
**2011-2012**	2	67.7	1	33.3	0	0	0	0	0	0	0	0	3	100
**Total**	641	75.9	32	3.8	94	11.1	13	1.5	34	4	30	3.5	844	100

Of 844, 641(75.9%) were considered as imported cases from other countries mainly from Afghanistan and 94 (11.1%) patients were recorded as introduced cases. Furthermore, 726(86%) of positive cases were male while 103(12.2%) were female and gender of 15 (1.8%) patients were not recorded. The mean age of the patients was 26.1 ± 11.33 years. The highest positive rate (39.4%) was seen in 21-30 year old group (305 cases) followed by 11-20 (28.3%) and 31-40 (17.4%) year old groups (Table 3). During 1997 to 2004, the number of malaria cases among 21-30 (from 10 to 58) and 31-40 (from 18 to 60 cases) year old groups gradually increased and then rapidly decreased. 203 (24.1%) cases were Iranian and 641 (75.9%) were immigrant/foreign born mainly from Afghanistan.

**Table 3:** Nationality, age and gender of malaria cases reported in Mazandaran Province, Iran, 1997-2012

Nationality	Age (yr)/Gender																	
	0-10		11-20		21-30		31-40		41-50		51-60		61-70		>70		Total	

	F	M	F	M	F	M	F	M	F	M	F	M	F	M	F	M	F	M
**Iranian**	6	8	17	36	14	39	13	21	8	12	3	5	1	3	0	2	62	126
**Immigrant**	6	24	10	156	9	243	5	96	2	25	3	5	0	1	0	1	35	551
**Total**	12	32	27	192	23	282	18	117	10	37	6	10	1	4	0	3	97	677

**N.B**. data on age and gender of 774 out of 844 cases were correctly recorded.

**Fig. 2:**
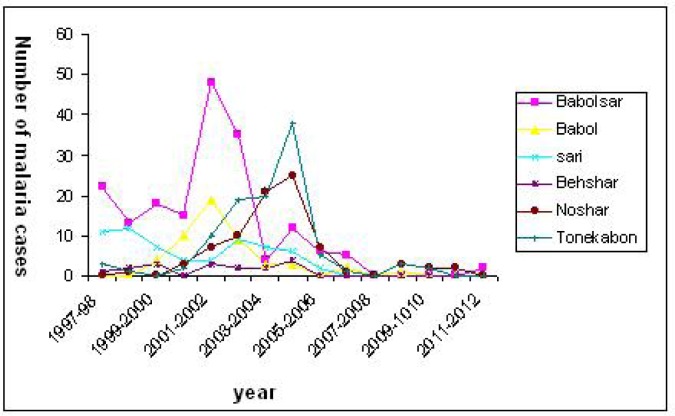
The number of malaria positive cases in relation to the cities located in the west, center and east of (two cities each ) Mazandaran Province, Iran, 1997-2012

Figure 2 showed that most of the malaria cases were registered at the Babolsar health care center (180 cases) followed by Tonekabon (107 patients) and Amol (102 cases).

Fewer cases were observed in Ramsar and Savadkoh (2 cases in each city). These results revealed that the number of malaria cases increased in 2001-2002 and 2004-2005 in most of the cities. The increasing of malaria incidence rate during 2001 to 2004 was particularly observed in cities located at west of the province excepting Ramsar. In addition, malaria cases were not reported in some cities among Mazandaran Province such as Babol and Sari during last three years.

## Discussion

According to malaria report in 2011, Iran located in area with low, geographically limited malaria transmission and effective malaria programmes but it is in neighborhood of countries which placed in area with high malaria transmission in the WHO Eastern Mediterranean Region such as Afghanistan and Pakistan. The majority of cases in Afghanistan and Pakistan, and almost all cases in the Iran are due to *P. vivax* (12).

The current study demonstrated that the most prevalent *Plasmodium* species was *P. vivax* (97.3%) among of malaria patients. These findings are in agreement with other studies and WHO-world malaria report (6, 7, 12–14). Our findings showed that the ratio of the mean number of positive slide to the mean number of examined slide was 0.18% in Mazandaran during 1997 to 2012. It showed significant decrease in comparison with results obtained from a study (0.22%) which was preformed in 1986-1997 (6). This result is in agreement with findings obtained from other part of the country (3, 14). For instance, the total malaria cases in the entire country were 96,340 with 45% *P. falciparum* in 1991 which gradually decreased to 18,966 with 12% *P. falciparum* in 2005 (3). In addition, this study demonstrated that the number of positive cases was low and similar during 1997 to 2000 but it was increased during 2001-2005 and then suddenly decreased to 40 cases in 2005-2006 and gradually decreased to 3 in 2011 (Table 1). These data indicated that effective programs for prevention and control of malaria performed in Mazandaran as well as the entire country which lead to be in the elimination stage in 2011(12, 15). However, increasing in malaria incidence rate in 2001-2005 could be due to immigration of Afghan people as well as immigration from malaria endemic region of Iran to Mazandaran. Data obtained from Statistical Center of Iran revealed that 199,508 persons emigrate to Mazandaran during 1995 to 2005 (16) which confirme this explanation. Also, about 75.7% of malaria patients were immigrant/foreign born mainly from Afghanistan. This outcome was significantly higher than amount obtained from last decade that indicated 25% of malaria patients were non-Iranian origin (6). This result is also comparable to a study conducted in other parts of Iran or other reports. For example, in Khorasan Razavi and Hormozgan provinces, 40% and 20.7 % of malaria cases were immigrants, respectively (14, 17). Furthermore, it is in contrast with a study from Altamim Province of Iraq which demonstrated that the highest cases were infected locally and not due to immigration (18).

On the other hand, the rising or decreasing rate of malaria patients was different in the cities in different years particularly in where located in the west of the province (Tonekabon, Noor and Noshar) in comparison with Babolsar which placed in the center (Fig. 2). The number of immigrants to Tonekabon and Noshar was higher than to other cities such as Ramsar and Neka which may explain this outcome (16). Furthermore, any malaria cases were reported in some cities including Babol, Sari, Behshar and Ramsar in the last three years indicating some parts of this province are malaria-free and in the prevention of re-introduction stage.

Number of relapse cases was 30 (3.7%) indicating of improper treatment of the infected cases (Table 2). Moreover, the highest infected rate was seen in age group of 21-30 years and then in 11-20 and 31-40 years. According with these data, the majority of the patients were male (87%). These findings are in agreement with results obtained from many studies which revealed that the most of malaria cases are male and young people, the potentially working groups, are the population groups at highest malaria risk (6, 13, 14, 17, 19).

In conclusion, these findings demonstrated that the trend of malaria, in Mazandaran in 2005-2012, follow the manner of malaria in other parts of Iran (elimination stage) and the most malaria endemic countries. The main reason of this great achievement is due to the surveillance of health system in disease control and the improvements in diagnosis and treatment. It also revealed that different regions among this province are in various stages of malaria control incidence. Moreover, this study suggested that malaria control strategy should be continued to Mazandaran become malaria-free region and in prevention of re-introduction stage.
